# A Review: Per- and Polyfluoroalkyl Substances—Biological Degradation

**DOI:** 10.3390/toxics11050446

**Published:** 2023-05-09

**Authors:** Dijana Grgas, Ana Petrina, Tea Štefanac, Drago Bešlo, Tibela Landeka Dragičević

**Affiliations:** 1 Faculty of Food Technology and Biotechnology, University of Zagreb, Pierotti Str. 6, 10000 Zagreb, Croatiatstefanac@pbf.hr (T.Š.); 2 Faculty of Agrobiotechnical Sciences Osijek, Josip Juraj Strossmayer University of Osijek, Vladimira Preloga 1, 31000 Osijek, Croatia; drago.beslo@pfos.hr

**Keywords:** perfluoroalkyl, polyfluoroalkyl, biodegradation, bacteria, fungi, enzymes

## Abstract

Per- and polyfluoroalkyl substances (PFASs), highly stable synthetic organic compounds with multiple carbon-fluorine bonds, are emerging as environmental contaminants, toxic, bioaccumulative, and environmentally persistent. PFASs are strongly resistant to biological and chemical degradation, and therefore PFASs present a challenge to researchers and scientists for a better understanding and application of remediation methods and biodegradation of PFASs and have become subject to strict government regulations. The review summarizes the recent knowledge of bacterial and fungal degradation of PFASs, as well as the enzymes involved in the processes of transformation/degradation of PFASs.

## 1. Introduction

The non-specific and general name “fluorinated substances”, which are also called “fluorochemicals” and “fluorinated chemicals”, represents a group of organic and inorganic compounds that contain at least one F atom and which are characterized by very diverse physical, chemical, and biological properties [1]. The Organisation for Economic Co-operation and Development (OECD) defined per- and polyfluoroalkyl substances (PFASs) as “PFASs are defined as fluorinated substances that contain at least one fully fluorinated methyl or methylene carbon atom (without any H/Cl/Br/I atom attached to it), i.e., with a few noted exceptions, any chemical with at least a perfluorinated methyl group (–CF_3_) or a perfluorinated methylene group (–CF_2_–) is a PFAS”. Both a perfluorinated methyl group and a perfluorinated methylene group are saturated and aliphatic. Note that the carbon in an R–CF_2_–O– or R–CF_2_–Si– group (R ≠ H/Cl/Br/I) is a perfluorinated methylene carbon. A perfluorinated methylene group may also be represented as “>CF_2_”, where “>” denotes two single bonds. A fully fluorinated carbon that is bound to the rest of the molecule by a double bond is a perfluorinated methylidene carbon atom (=CF_2_). This distinction is important. Further, a perfluorinated methane carbon moiety (>CF–) alone does not meet this revised PFAS definition” [2]. Per- and polyfluoroalkyl substances can be classified into three groups: perfluoroalkyl substances, polyfluoroalkyl substances, and fluorinated polymers [3,4]. Polymeric PFASs, such as branched fluorinated polymers, fluoropolymers, and polymeric perfluoropolyethers, are macromolecules that contain repeating monomers [5,6]. PFASs can form linear and branched structures, and the formula C_n_F_2n+1–_ includes both linear and branched structures [3,7]. The alkyl chain of perfluoroalkyl substances, i.e., perfluoroalkyl-substituted compounds, is fully fluorinated. Perfluoroalkyl substances include (aliphatic) perfluorocarbons (PFCs), perfluoroalkyl acids (PFAA), perfluoroalkane sulfonyl fluorides, perfluoroalkane sulfonamides, perfluoroalkyl iodides, perfluoroalkyl aldehydes [3]. The alkyl chain of polyfluoroalkyl substances is partly fluorinated and contains one or more fluorine atoms, but not all H atoms are replaced by F atoms [3,4]. Polyfluoroalkyl substances include perfluoroalkane sulfonamide derivates, fluorotelomer-based compounds, semifluorinated *n*-alkanes, and alkenes [3]. 

PFASs are persistent in the environment [1] as a whole, and PFAS precursors can be degraded to perfluoroalkyl sulfonic acids (PFSAs) and perfluoroalkyl carboxylic acid (PFCAs) [8]. PFASs are permanently introduced into the aquatic ecosystem, and organisms present downstream of the release are continuously exposed to PFASs [4]. The three crucial characteristics that are used for the management of commercial chemicals in the area of environmental protection are bioaccumulation, persistence, and toxicity [9], with persistence being the most important. Without persistence, the potential toxicity and bioaccumulation of the chemical are less significant [10].

Since PFASs were found to be persistent in the environment and some of them toxic contaminants [1,11,12,13,14,15,16,17,18,19,20], strict legal regulations were set [3,21,22,23], together with industry actions, in order to decrease the amount of long-chain PFCAs, PFSAs, and their precursors that end up in the environment [11]. Depending on the circumstances of the exposure (route of exposure, magnitude, and duration, etc.) and factors associated with exposed individuals (e.g., ethnicity, health status, genetic predisposition, age, gender), PFASs can potentially produce a wide range of adverse health effects [12]. Perfluorooctanoic acid (PFOA) and perfluorooctane sulfonate (PFOS) are the most commonly detected compounds in the highest concentrations in environmentally exposed organisms, with multiple toxicities reported in experimental models, wildlife, and humans [12,17]. 

The information regarding microbial PFAS transformation/degradation is limited [10,14,24,25,26,27,28,29,30,31,32]. Complete mineralization of PFASs is not expected under natural conditions [24] or in conventional biological processes because of the high strength C-F bond [28]. It was reported that there are several particular structural combinations that may result in specific groups of PFASs that can be mineralized [1], such as novel fluorosurfactants [33,34,35,36]. However, structural combinations that make non-persistent PFASs are scarce [1]. Polyfluoroalkyl substances, more than perfluoroalkyl compounds, are prone to microbial attack [14]. The stability of key chemical bonds, such as esters, ethers, etc., is important because the overall stability of PFASs depends on them [24]. Wei et al. [5] suggest that PFAS biodegradation depends on the properties of the compound, such as the carbon chain length and the microorganism type. The biodegradation of PFASs is usually determined by determining products of biodegradation and by determining the kinetics of degradation by chemical analysis; because PFASs have a low oxygen demand and do not undergo complete mineralization, so methods based on the CO_2_ determination, dissolved organic carbon and biological oxygen demand (BOD) cannot be applied. As defluorination occurs during the biodegradation of PFASs, measuring the concentration of fluoride ions can provide insight into the biodegradation of PFASs, and thus the extent of defluorination can be determined [24]. Joudan et al. [37] highlighted the challenges during the identification of transformation products of PFASs, by reviewing the results of scientific experiments of biotransformation in microbial systems (under aerobic or anaerobic conditions in soil samples, soil from a site contaminated with PFASs, soil-plant microcosmos, and sludge from the wastewater treatment plant), model animals (in vivo studies with rats or fish), or in vitro using materials from animals or humans (subcellular liver fractions, microsomes, cytosol and hepatocytes, and also specific enzymes such as cytochrome P450 isozymes or alkaline phosphatase for polyfluoroalkyl phosphate esters). Limited PFASs water solubility usually requires small amounts of co-solvents (to prevent toxicity to the biological system) when introducing a test chemical to the experimental system, such as acetonitrile. An alternative to organic solvents could be passive dosing. The authors suggest fluorine measurements to obtain the mass balance of PFAS degradation due to the detection of unidentified transformation products. Since the perfluoroalkyl moieties lack polarizability, the PFASs have increased volatility in comparison to their hydrogenated counterparts, so sampling of volatile analytes should be performed from the experimental headspace. An additional challenge is the fact that most commercial PFASs are created as mixtures which complicate intermediate and product appropriation since the individual species in the mixture are often structurally related, and the experiments of PFAS biotransformation would be simpler with commercially available pure chemicals. They encourage using a variety of techniques, including gas chromatography and liquid chromatography in both positive and negative ionization mode, and high resolution mass spectrometry as needed for the detection of unknown biotransformation products, as well as reactive and transient intermediates. The authors highlighted the use of both negative and positive controls. They point out that the short-chain PFAS analysis is challenging due to their water solubility resulting in poor retention on reversed-phase HPLC columns [37]. Microbial degradation and enzymatic treatment could provide environmentally friendly, sustainable, and cheap possible alternatives for PFAS bioremediation.

This review provides an overview of the current knowledge about PFASs biodegradation and enzymes in PFASs transformation.

## 2. Chemical Stability of PFASs in the Environment

PFASs are characterized by a very stable and strong C–F bond, thermal and chemical stability, and their lipophilic and hydrophobic nature, which is why surfactants and polymers that have a perfluoroalkyl group have enduring and useful properties [38]. The C–F bond is very strong due to the small size and very high electronegativity of the fluorine atom [39], which makes the PFASs highly resistant to degradation by oxidative/reductive processes. The low polarizability of fluorine atoms is the reason for weak intermolecular interactions. The presence of acid functional groups makes numerous PFASs strong acids [40]. The protonated and anionic form of PFASs significantly affects their physical and chemical properties (Henry’s law constant, vapor pressure, octanol/water partition coefficient), with fully dissociated anionic forms of PFASs being frequently present at pH values relevant to the environment. The octanol/water partition coefficient increases with the molecular weight or length of the C-chain, and it mostly refers to the acid form [41], so the values of the octanol/water partition coefficient are not relevant to the anionic form [7]. Chain length and functional groups give unique physicochemical properties to per- and polyfluoroalkyl substances [13]. Perfluoroalkyl-substituted compounds are poorly soluble in water [24]. The perfluorinated carbon chain (C_n_H_2n+1_) is hydrophobic and functional groups (–SO_3_^−^ and –COO^−^) are hydrophilic [42]. Long-chain PFSAs (C_n_F_2n+1_SO_3_H, n ≥ 6) and PFCAs (C_n_F_2n+1_COOH, n ≥ 7) and their anions, in comparison to their short-chain analogs, bioaccumulate and bioconcentrate significantly more [3] and they are recognized as persistent and toxic contaminants [11]. Perfluorooctane sulfonic acid (PFOS) and related compounds are recalcitrant due to the strong C–F bond, the perfluoroalkyl chain rigidity, and the absence of the reactive substituents in the PFAS molecules [38].

### 2.1. Production of the PFAS

Production of PFASs has been going on for 70 years, and polymers and surfactants derived from PFASs have wide commercial and industrial applications [38] due to their amphiphilic properties [3,38]. PFASs are widely used in the military, numerous industrial, residential, and commercial spheres, such as surfactants in the production of fluoropolymers, metal coatings, aqueous film-forming foams (AFFFs) and paper products, household and textiles [3,11,25,26,38,43,44,45]. The application of surfactant is in the production of AFFFs, which are used to extinguish highly flammable liquids, coatings, and auxiliary means for the production of fluoropolymers. Polymers have applications in soil repellents, textile dyes, and grease-resistant food-grade paper [3].

### 2.2. Occurrence in the Environment

The widespread use of PFASs has resulted in PFASs being found in humans, wildlife, and the environment [3,12], with the potential for long-range transport [11]. Humans can be exposed to PFASs in many ways, such as over drinking water, food (such as fish), consumer products, and indoor air/dust [46]. PFASs have been found, for example, in food, breast milk, drinking water, air, airborne dust, etc. [4,47]. Although a reliable estimation of the production of PFASs, direct and indirect emissions of PFASs into the environment is not known [4], the vast majority of PFAS emissions, over 95%, are directly released into the aquatic environment and the emission of PFASs through the atmosphere has a small contribution to the overall emission, less than 5% [45]. PFASs have shown the potential for long-range transport through the water and atmosphere [48,49], and based on their physicochemical properties and environmental conditioning, PFASs undergo diverse transport, partitioning, and degradation processes [50]. One of the most significant sources of PFAS emissions is municipal landfills [5] and industrial and municipal wastewater treatment plants [51] because the applied wastewater treatment technologies are not efficient enough for the removal of micropollutants [52]. Advanced water treatment methods, such as nanofiltration and activated carbon, could contribute to the reduction of PFAS concentrations [40,53]. 

Currently, a substitution at the industrial level of long-chain PFSAs, PFCAs, and their precursors with suitable alternatives, especially other PFASs like homologs that have shorter chains and perfluoropolyethers (PFPEs) in applications where extremely low surface tension and/or long-term water and oil repellency is required is in progress. However, an issue arises whether these fluorinated alternatives are less harmful to the environment and people than their predecessors. Authors furthermore suggest that most alternatives (GenX, PFTECA_1_, PFTECA_2_, 6:2 FTCA (an alternative to PFOA) F-53, F-53B, PFBSaPa (an alternative to PFOS), 5:1, 3:1 FTOHs (alternative to 8:2 FTOH) (fluorotelomer alcohol), Forafac, EF-N, Novec, degradation products of RM720, PFOTSi and its degradation products (alternative for certain POSF- and/or fluorotelomer-based compounds)) do not differ substantially from PFOS, perfluorooctanoic acid (PFOA) and their precursors in terms of physicochemical properties and likely fate in the environment. Their results suggest that most alternatives will be similarly persistent and mobile in the environment as long-chain PFASs, so it is likely that fluorinated alternatives will become as widespread as their predecessors [11]. 

The most detected PFAS in aquatic systems and drinking water worldwide are PFOS, CF_3_(CF_2_)_7_SO_3_H, and PFOA. They have been found in activated sludge, sediments, landfill, and soil [5]. Since the production and direct release of PFOS and PFOA into the environment have been stopped, the determination of level of exposure and effect of PFASs is determined by measuring PFOS, PFOA, and other PFAA into which various PFASs have been converted. Thus, efforts have been made to investigate what amounts of PFCA, C_n_F_2n+1_COOH, and PFSA, C_n_F_2n+1_SO_3_H, with an emphasis on PFOA and PFOS, originate from the cleavage of non-fluorinated groups, namely perfluoroalkyl and polyfluoroalkyl groups in the environment [24].

Polyfluoroalkyl compounds can undergo abiotic or biotic transformations and eventually form perfluoroalkyl-substituted compounds in the environment, so the polyfluoroalkyl compounds can be considered precursors of perfluoroalkyl-substituted compounds [27]. Similarly to the bioaccumulation potential of PFASs, the structural properties of PFASs affect their environmental fate to a large extent [25]. 

The final stage of PFASs, in the life cycle of PFASs, is landfills, since PFASs are released from wastes containing PFASs [54]. So, PFASs were detected in the landfill leachate, with PFCAs and PFSAs being the most frequently detected [55]. Not only that, but PFASs can end up in the surrounding soil and water systems by leaching from landfill leachate [44]. The waste at the landfill undergoes aerobic, acetogenic, and methanogenic phases, which make physiochemical changes in waste and leachate, including changes in pH value and organic and inorganic compounds [56]. The release of the majority of the PFASs from waste to leachate happens due to biodegradation, more specifically, the start of the methanogenetic phase. A pH value of the leachate higher than 7 during the methane-yielding stage contributes to larger perfluoroalkyl acids (PFAAs) mobility [44], and the reason could be the changed electrostatic behavior of the sorbents [57]. It was found that the sorption of PFOA and PFOS decreased as the pH of the solution increased because of protonation of the adsorbent surface, which resulted in less positive sites on the sorbent [58,59]. The concentration of PFAA in leachate was negatively [60] and positively [61] correlated with electrical conductivity. Sorption behavior is connected to the carbon chain length, the functional head group of the PFASs [57], multivalent cations [62], and anions [42]. Leachate can be treated by physiochemical or biological methods. Leachate is commonly treated at off-site domestic wastewater treatment plants [44]. There is also on-site pretreatment, followed by off-site discharge at a wastewater treatment plant and complete treatment and discharge on-site [63]. 

The short-chain PFAAs (C4-C7) are predominant, more soluble, and more persistent in water than long-chain PFAAs, so the short-chain PFAAs are more likely to leach out from solid wastes [64]. During the period 2009 to 2017, in the aquatic environment, 455 PFASs were detected in the form of neutral molecules, cations, anions, and zwitterions [65]. Throughout their whole life cycle PFASs, are released into the aquatic environment [4]. Volatile PFASs, such as fluorotelomer alcohols, perfluoroalkyl sulfonamides (PPOSA), perfluoroalkyl sulfonamidoethanols that have been found in outdoor air samples enter the atmosphere, where their degradation can occur, and formation of intermediates during atmospheric oxidation, or transformation into PFASs that are more persistent, such as PFCAs and PFSAs, and eventually reach the aquatic environment [4]. Due to the volatile to semivolatile properties of the PFAS precursors (fluorotelomer alcohols, perfluoroalkyl sulfonamides, perfluoroalkyl sulfonamidoethanols, polyfluoroalkyl phosphoric acid esters), they are usually transported via the atmosphere and then can be degraded, among others, to PFCAs and PFSAs [66,67]. The PFAS precursors undergo many transformation pathways in the atmosphere or under aerobic and anaerobic conditions in other environmental compartments [67,68]. The final degradation products are then mostly transported via the water phase and, to a lower extent, via seaspray or gas-phase and particle-bound transport in the atmosphere [48,69,70]. For remote regions, the gas-phase transport is probably the dominant pathway for neutral, volatile PFASs, and for ionizable PFASs, the dominant transport pathway is speculated to be atmospheric transport or transport via the water phase [51]. The physicochemical properties of the PFASs and environmental conditions affect the environmental cycling of PFASs [43,71]. While the short-chain PFASs are mostly more mobile in hydro systems due to hydrophilic properties, long-chain PFASs have bioaccumulation potential because they bind to particles due to hydrophobic properties [43,72]. Therefore, PFASs are dominantly present in sediment and oceans [73]. 

The PFAAs can enter the soil through atmospheric wet deposition, the use of AFFFs, and landfill [29]. Short-chain PFASs are less strongly retained in soils than long-chain PFASs, and short-chain PFASs can more rapidly transport and infiltrate into the deeper soil and groundwater. Long-chain PFASs are usually retained in shallower surface soil. In soils, PFASs with a negative charge are more mobile that PFASs with a positive charge or neutral ones, so they can quickly enter the groundwater. Since PFASs are persistent and slowly desorbed, PFASs that are even more strongly sorbed can be a sustained source of the pollution of groundwater [74]. 

### 2.3. Toxicological and Ecological Effect of the PFAS

The bioaccumulation potential of PFASs varies among individual organisms and species and depends on the physicochemical properties of PFASs, such as chain properties (straight or branched), functional groups, and chain length [43]. Perfluoroalkyl-substituted compounds, the ones with only C–F bonds, are usually more toxic and persistent in the environment in comparison to polyfluoroalkyl compounds [12,27]. Accumulation of PFAS in the human body and exposure to PFASs has been connected to many detrimental effects on health, and some of them are pancreatic tumors, reproductive and developmental deficits, neurotoxicity, immunotoxicity, and hepatomegaly and hepatic peroxisome proliferation [4,12,17]. The animal toxicity tests revealed persistent growth deficits, compromised postnatal survival, and development delays [18,19,20].

Long-chain PFCAs and PFSAs have caused concern due to possible toxicological and ecological effects with numerous international mechanisms since 2000. having been undertaken by industry and regulatory bodies to reduce the presence of long-chain PFCAs, PFSAs, and their precursors in the environment, as well as their release into the environment [3,11]. Bioaccumulation of PFOA in biota has not been found [27]. The approximate half-life of PFOS in water is 41 years [75], and in humans, 3 years for PFOA and 5.4 years for PFOS [76]. In accordance with the EPA guidelines, PFOA and PFOS health effects assessments have been conducted for human health [77] and showed a probable correlation between PFOA exposure and pregnancy-induced hypertension, testicular and kidney cancer, ulcerative colitis, thyroid disease, and high cholesterol [78], and a notable concern for adverse effects of the PFOS on the human immune system due to the observed lower antibody titers associated with the exposure levels of PFOS [79].

## 3. Bacteria in Biodegradation/Transformation of PFASs 

Microbial communities can provide important information: the toxicity of PFASs to microorganisms and the degradation or transformation of PFAS constituents by microorganisms [25]. High concentrations of PFASs did not show toxicity to microbial activity during the biodegradation of PFASs [24]. To test the biodegradability of PFASs, activated sludge from biological wastewater treatment plants is often used as a test medium [31] or as an inoculum [32]. Wang et al. [32] achieved biodegradation of 6:2 fluorotelomer sulfonic acid (6:2 FTSA, C_6_F_13_CH_2_CH_2_SO_3_H) with activated sludge from one biological wastewater treatment plant (WWTP), but not with activated sludge from another plant. Additionally, it has been shown that the activated sludge has the capacity to adsorb PFOA in the range of <5.0–69.0 mg/g and PFOS in the range of 13.1–702.2 ng/g [80]. The conventional activated sludge process in WWTP can be used for landfill leachate [5], but biological processes were not successful in PFAS degradation [81,82]. Additionally, for the treatment of landfill leachate, membrane bioreactors (MBRs) have been usually employed, but the PFAS removal from landfill leachate was not efficient [83]. The combination of physicochemical and biological methods could be a promising way for the achievement of satisfactory effluent values of leachate, for instance, the biological treatment and nanofiltration or reverse osmosis or activated carbon [5]. Aerobic bacteria have been found to transform some PFASs, such as 8:2 FTOH [84], 6:2 fluorotelomer sulfonate (6:2 FTS) [32], N-ethyl perfluorooctane sulfonamidoethanol (N-EtFOSE) [31], 6:2 fluorotelomer alcohol (6:2 FTOH) [85]. It was suggested that the probable mechanisms of aerobic biodegradation of fluorinated organic compounds involve catalysis by cytochrome P450 monooxygenase, transaminase metabolism, and beta-oxidation [32,84,85,86]. Anaerobic bacteria *Pseudomonas* strain D2 under sulfur-limiting conditions partially degraded sulfonates, like H-PFOS and 2,2,2-trifluoroethane, with hydrogen by defluorinating them [87]. *Acidimicrobium* sp. strain A6 has been reported to transform PFOA and PFOS in mg/L level concentration in well-controlled laboratory experiments [88].

O’Carroll et al. [25] investigated in situ microbial communities and groundwater samples in the area polluted with AFFFs. Their results suggest PFAS degradation, especially 6:2 FTS degradation. Additionally, their results showed the relative abundance of over one hundred individual genera, which were significantly associated with PFAS chemistry. A strong positive correlation was observed between the lineages within the *Desulfococcus* genus and PFASs, and a strong negative correlation between the Oxalobacteraceae family and PFASs. Furthermore, the authors suggest that some genera might have been inhibited at high PFAS concentrations, and a range of genera (*Acidimicrobium* and *Gordonia*) might have been simulated at low to mid-range concentrations [25].

The aerobic bacteria *Pseudomonas aeruginosa*, strain HJ4, was reported to be capable of PFOS biodegradation [89]. The experiments of the PFOS biodegradation were performed with a range of PFOS concentrations (1400–1800 µg/L). After 48 h of incubation, PFOS was decomposed by approximately 67%, and the residual concentration of PFOS was constant. The *Pseudomonas aeruginosa* was isolated from wastewater treatment sludge from a municipal biological wastewater treatment plant. The incubation was performed with the addition of 0.1% (volume) of glucose. The glucose was not essential, but it supported the growth of the HJ4 strain. The concentration of the fluoride ions was monitored by ionic chromatography, but no evidence of defluorination of PFOS was found, both in samples incubated with *Pseudomonas aeruginosa* and in abiotic controls. Products PFBS and PFHxS (perfluorohexane sulfonate) were detected in the range of concentrations of 4.0–26.0 ng/L. Additionally, tandem MS (Mass Spectrometry) analysis showed the formation possibility of several unknown products. The authors suggest the partial cleavage of C–C bonds in the PFOS molecule instead of C–F bonds [89].

Activated sludge from full-scale municipal WWTP after the activated sludge treatment was responsible for the lowered concentration of the shorter-chain PFASs (PFHxA (perfluorohexanoate) and PFHxS), which points to the fact that microorganisms from activated sludge may degrade more hydrophilic and mobile PFAS of shorter carbon chains [83]. In the same activated sludge process, PFCAs, PFSAs, and PPOSA increased due to their precursor compounds’ biodegradation [82].

Ochoa-Herrera et al. [14] investigated microbial toxicity and susceptibility to biodegradability of PFOS and shorter-chain perfluoroalkyl and polyfluoroalkyl sulfonic and carboxylic acids. They used several sources of inoculum: activated sludge from various WWTP receiving municipal wastewater or PFOS-containing wastewater, anaerobic granular sludge, anaerobically digested sewage sludge, and sediments. Toxicity to the methanogenic activity of anaerobic wastewater sludge of all tested compounds (PFOS, 6:2 FTSA, PFBS, TFA (trifluoroacetic acid), and C_3_- and C_5_-PFAS compounds) was not found even at as high concentrations as 500 mg/L. All investigated PFASs showed high resistance to microbial degradation. Ochoa-Herrera et al. [14] suggest the sequenced aerobic and anaerobic degradation for the mineralization of PFOS and related PFASs.

Ochoa-Herrera et al. [90] reported about 18% defluorination of technical PFOS and 71% defluorination of PFOS branched isomers by reductive dehalogenation of PFOS in combination with vitamin B_12_ as a biocatalyst and Ti(_III_) citrate as a bulk reductant. There are also some other examples of aerobic degradations, such as (6:2 FTSA, F(CF_2_)_6_(CH_2_)_2_SO_3_H) and 8:2 FTOH (F(CF_2_)_8_(CH_2_)_2_OH), where multiple –CF_2_—groups have been removed and formed shorter chain perfluorocarboxylic acids and CO_2_ [84,85,87].

The biodegradation of polyfluorocarbon chains is initially centered on the hydrocarbon functionality to which a polyfluorocarbon moiety is attached [91]. The additional functionality (chlorines, phenyl rings, sulfonates, carboxylates, or phosphonates) provides a weak entry point for reactions that activate C–F bonds and lead to their eventual cleavage. When additional functionality is attached to a highly fluorinated, even perfluorinated, chain or ring, there are other chemical and metabolic possibilities available to achieve defluorination [92].

Contrary, Liou et al. [10] found no evidence of microbial metabolism of perfluorooctanoic acid (PFOA). They performed experiments with five different microbial communities (from industrial site sediment, a municipal WWTP, soils from two fire training areas, and agriculture soil). During up to 259 days of anaerobic incubation with PFOA (concentration of 100 ppm and 100 ppb) as an electron acceptor and acetate, lactate, ethanol, and/or hydrogen gas as electron donors, the authors found no evidence for a reductive defluorination process with naturally occurring microbial populations. They also examined the potential cometabolism of PFOA at 100 ppm and 100 ppb concentration during reductive dechlorination of TCE (trichloroethene) with 5% inoculum of sewage treatment plant as an electron acceptor over 65 days of incubation. However, reductive dechlorination-based cometabolism of PFOA could not be confirmed. Not only that, the authors investigated a potential transformation of the PFOA (electron donor) (100 ppm and 100 ppb) with five electron acceptors: aerobic, nitrate reduction, iron reduction, sulfate reduction, and methanogenesis with a 5% sewage treatment plant inoculum, with and without acetate as an electron-donor, during 110 days of incubation. Nevertheless, no significant changes in PFOA concentration or fluoride concentration were found in any of the experiments [10]. Schultz et al. [83] investigated the flourochemical mass flow of the most abundant perfluoroalkyl sulfonates, perfluoroalkyl carboxylates, fluorotelomer sulfonates, and fluoroalkyl sulfonamides in a municipal wastewater treatment plant that was composed of the screen, primary clarifier, trickling filters, activated sludge aeration basins, and secondary clarifier. A decrease in the mass flows was recorded for perfluorohexane sulfonate, perfluorodecanoate and perfluorohexanoate. The unchanged mass flows for 6:2 FTS fluorotelomer sulfonate and perfluorooctanoate were reported. The increase in the mass flows for perfluorooctane, perfluorodecane sulfonates, perfluoroalkylsulfonamides, and perfluorononanoate was observed [83].

## 4. Fungi in Biodegradation/Transformation of PFASs

The most used fungi for the degradation of toxic contaminants are brown-rot fungi *Aspergillus niger* and the white-rot fungi *Phanerochaete chrysosporium*. *P. chrysosporium* produces lignin peroxidases (LiP) and manganese peroxidases (MnP), and *A. niger* dioxygenase, xylanases, cellulases, and hemicellulases [27]. It has been shown that the remediation and treatment of PFASs, as well as the effective decomposition of PFOA, is feasible using ECOHR (enzyme-catalyzed oxidative humification reaction) [93]. ECOHR reactions are commonly present in the soil system and characterized by a series of oxidative reactions during the humification process. ECOHRs are carried out by natural extracellular enzymes secreted by white and brown rotting fungi, namely laccases (LaC), LiP, MnP, etc [94]. These enzymes degrade PFASs by first converting chemical compounds from nature into reactive radicals, which then react with the PFAS (an inactive molecule) [95]. Colosi et al. [93] conducted PFOA degradation experiments using horseradish peroxidase (HRP), hydrogen peroxide, and 4-methoxyphenol as a cosubstrate. Over 6 h, they recorded a 68% degradation of the parent compound and a 98% reduction in acute aquatic toxicity. To analyze the concentration of PFOA and 4-methoxyphenol, they used HPLC (high-performance liquid chromatography). To determine fluoride ions, they used ion chromatography (IC). By-products were determined using GC-MS (gas chromatography-mass spectrometry). To determine toxicity, they used the Microtox toxicity testing system. In the experiments without cosubstrate, no removal of PFOA was recorded. The authors believe that the radical intermediates formed in the reaction between 4-methoxyphenol and HRP are responsible for the non-specific degradation of PFOA by cleaving the C–F bond in PFOA. Additionally, the authors suggest that the degradation of perfluorinated aliphatic compounds occurs via one of two mechanisms: (i) radical action on the double bond followed by fermentation and (ii) Kolbe decarboxylation followed by stepwise conversion of –CF_2_ units to CO_2_ and fluoride ions [93].

Luo et al. [30] also used ECOHRs for PFOA degradation using laccase and achieved 50% PFOA degradation over 157 days in the presence of 1-hydroxybenzotriazole and a mineral buffer solution. In the experiments, they used laccase from *Pleurotus ostreatus*. Using HR-MS (high resolution mass spectrometry) analysis, they selected decomposition products of fluorinated alcohols and aldehydes with shorter carbon chains, and the pseudo-first-order rate constant was 0.0044 day^−1^ with r^2^ = 0.89. In these experiments, they did not detect PFCAs with shorter carbon chains. These authors suggest that the transformation of PFOA could take place during humification [30]. Contrary, a 40% degradation of PFOA was achieved during 140 days in the presence of soybean meal and laccase in a soil slurry [29].

Previous research on the aerobic biotransformation of perfluoroalkane sulfonamido derivatives has been conducted on compounds that have eight perfluorinated C atoms, with an emphasis on EtFOSE, which is ultimately degraded to PFOS [3]. Some polyfluoralkyl compounds (commercial surfactants, AFFF components, and polymers composed of acrylate-linked fluorotelomers) have labile functional groups that allow some transformations in the environment that mostly end up with the formation of terminal PFSA and PFCA degradation products [37]. However, during that degradation, reactive intermediates are generated that can be more toxic than the PFSA and PFCA terminal degradation products [15,16]. Tseng [27] investigated the degradation of PFASs (FTOH, PFOA and PFOS) by the ligninolytic fungi *Phanerochaete chrysosporium* and *Aspergilus niger* and by the oxygenase-expressing bacteria *Pseudonocardia dioxanivorans* CB1190, *Methylosinus trichosporium* OB3b, *Burkholderia cepacia* G4 and *Pseudomonas putida* F1, as well as five fungal strains and two strains of aerobic bacteria that were isolated from a site contaminated with AFFF. They reported a 50% transformation of 6:2 FTOH and a 70% transformation of 8:2 FTOH by *P. chrysosporium* over 28 days. Contrary, during 35 days, no transformation of 6:2 FTOH by *A. niger* was recorded. In PFOS degradation experiments using fungal isolates Envi 5 and Envi 7, about 20% transformation of PFOS was achieved during 28 and 14 days, respectively. During 7 days, no transformation of either PFOA or PFOS by bacteria was recorded [27].

Merino et al. [26] conducted fungal biotransformation experiments of 6:2 FTOH (C_6_F_13_CH_2_CH_2_OH), a PFAS compound; 6:2 FTOH is one of the shorter-chain substituents of 8:2 FTOH, and 8:2 FTOH is a precursor of long-chain PFCAs, such as PFOA [13,22]. When 6:2 FTOH is released into the environment, it can be transformed into PFCAs and other polyfluoroalkyl substances by means of biological and physicochemical processes [96]. Biotransformation of 6:2 FTOH has been investigated using bacteria and a microbial consortium [8] and fungal biotransformation using the wood decay fungus, *Phanerochaete chrysosporium* [97]. In relation to bacterial biotransformation, fungal biotransformation of 6:2 FTOH is more favorable for the remediation of products based on 6:2 FTOH due to the nature of biotransformation metabolites that are then more easily transformed in the environment [26]. Merino et al. [26] used two fungal strains of wood decay: *Gloephyllum trabeum* and *Trametes versicolor*, and six fungal isolates: TW1-3 (closely related to the genus *Fusarium* sp.), TW4-2 (closely related to the genus *Aspergillus* sp.), TW4-1 (closely related to the genus *Aspergillus* sp.), B76 (closely related to the genus *Fusarium* sp.), B78 (closely related to the genus *Penicillium* sp.) and B79 (closely related to the genus *Penicillium* sp.), from a location contaminated with PFASs, using AFFF. LC-MS/MS (liquid chromatography-tandem mass spectrometry) was used to determine the course of transformation of the initial compound and transformation products. *G. trabeum* and *T. versicolor* transformed 6:2 FTOH into nine and six quantifiable transformation products. All six fungal isolates achieved the transformation of 6:2 FTOH to 5-9 quantifiable transformation products during the 28 days of the experiment under the tested conditions at different molar removals. There is potential for media supplementation to improve 6:2 FTOH biotransformation rates and product profiles. They also investigated the tolerance of six isolates to PFOA and PFOS for 14 days, examining concentrations of 0-1000 mg/L. Six fungal isolates tolerated up to 100 or 1000 mg/L of perfluorooctanoic acid and perfluorooctane sulfonic acid, with some isolates showing growth with increasing concentrations [26]. 

Luo et al. [98] used laccase isolated from the fungus *Pleurotus ostreatus* to degrade PFOS over 162 days. When degrading PFOS, Luo et al. [98] applied ECOHRs induced with laccase, and they used HBT (1-hydroxybenzotriazole) as a mediator. ECOHR reactions are important in the natural process of humification [98]; they are catalyzed by extracellular phenoloxidases and peroxidases. Laccases are a group of phenoloxidases that can catalyze ECOHR [99]. HBT can be used as a mediator in the system where laccase is applied because it has a high reaction efficiency and low environmental impact and is commonly present in natural organic substances [100]. During decomposition, the fluoride concentration was determined by IC and the organic products were determined using HR-MS, Orbitrap Elite ESI-HRMS (Electrospray ionization-High-resolution mass spectrometry), and MS/MS. Fluoride release was faster in the system with Cu^2+^ than with Mg^2+^, and the defluorination ratio was 47.4% for Cu^2+^ and 47.1% for Mg^2+^. The authors believe that the degradation of PFOS is carried out by similar mechanisms but at different rates. The interaction between cations and PFOS was determined by differential UV-vis spectrometry. Density functional theory modeling showed that the formation of a complex between PFOS and metal ions could release its helical configuration and reduce the energy of the C-C bond in PFOS, which facilitates the attack of radicals on the C–C skeleton of PFOS. The authors suggest that the direct attack of HBT radicals on PFOS initiated a free radical chain reaction process and led to the formation of fluorides and partially fluorinated compounds. ECOHR could be a potential pathway for PFOS degradation in the environment, which could be a sustainable approach for the remediation of PFOS contamination through the addition of appropriate enzymes and mediators [98].

In addition to the type of activated sludge, the type of soil also affects the biodegradation of PFASs. Thus, microorganisms from the soil are used for the degradation of fluorotelomer compounds under aerobic conditions, and the results of the degradation were dependent on the different native microbial cultures that live in a particular soil, as well as on the chain length of the tested compound. For example, agricultural soil was not suitable for decomposition, but forest soil was suitable due to a higher concentration of fungal biomass [91]. The type of sediment, such as river sediment or marine sediment, contaminated by the fluorotelomers and in situ microbial community affects the PFAS degradation products [24,101]. Degradation outcomes of PFASs differ largely with the applied incubation matrix for the same compound [24].

**Figure 1:**
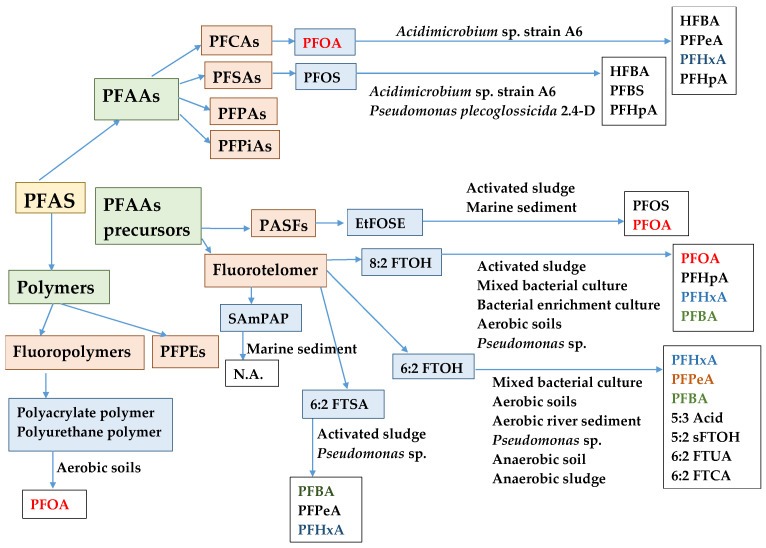
Types of microbes or microcosmos in biodegradation/transformation/defluorination of selected PFAS [10,24,31,32,74,84,85,86,88,102,103,104,105,106,107,108,109,110,111,112,113].

Research of PFAS biodegradation/transformation/defluorination was conducted under different operating conditions with different microorganisms (Figure 1) and resulted in different products and process efficiencies [24,74,102].

## 5. Enzymes for the Transformation of PFASs

Fungi employ oxidative mechanisms over extracellular and metal-containing oxidoreductases for the degradation of pollutants [27]. Oxidoreductases, laccases, soy peroxidase, HRP, LiP, MnP, and chlorine peroxidase are mainly used in the processes of biological degradation of new pollutants [30]. Fungi usually use MnP, LiP, LaC, cellulases, and hemicellulases [27]. Oxidoreductases include dehydrogenases, peroxidases, oxygenases, and oxidases. Laccases and peroxidases are most often used in remediation processes because they have the ability to break down a wide group of different organic contaminants [114], which is attributed to their ability to create radicals that break down the basic pollutant into smaller products that can be more easily biologically degraded and have minimal toxicity [115].

Degradation based on the use of enzymes for the degradation of target compounds offers advantages such as working at low or high concentration of pollutants, less sludge production, less energy required and can be applied to a wide range of pollutants [115]. On the other hand, during bioremediation, enzyme conformation may change due to unfavourable environmental conditions, catalysis is expensive, the enzyme cannot be reused, and harmful soluble by-products may be formed [116]. Such defects can be avoided by immobilizing the enzyme on supports and by insolubilizing the enzyme (such as crosslinking) [115].

### 5.1. Laccases

Laccases are produced by plants, insects, bacteria, and fungi. It is a group of multiple copper oxidases. Laccases produced by wood-decaying fungi show a high ability to oxidize various compounds, and they are characterized by a wide spectrum of substrate specificity [117]. Laccases typically have four copper ions: one T1, one T2, and two T3 copper centers. There are three types of laccases: (i) type 1 laccase—has one Cu atom, blue LaC, (ii) type 2 laccase, normal LaC—has one Cu atom; and (iii) type 3 laccase, fused dinuclear laccase—has two Cu atoms [118]. In laccase type 1, the copper ion is responsible for the biocatalytic effect of the oxidation-reduction potential, and this is where the oxidation of the substrate takes place. Microbial laccases have a stronger redox potential than plant laccases. Type 2 and 3 laccases form a trinuclear cluster (T2/T3) for the reduction of molecular oxygen to water by electron transfer from T1 to the trinuclear site [119]. In relation to the fact that peroxidases use hydrogen peroxide in the catalytic reactions carried out by laccases, the use of atmospheric oxygen as an electron acceptor is advantageous, which is why laccases are widely used for the degradation of numerous new pollutants and other undesirable persistent compounds [118]. Unlike peroxidases, such as LiP and MnP, which require a cofactor, laccases can degrade the target compound without an external source of Mn^2+^ or H_2_O_2_ [115].

Laccases can catalyze one-electron oxidation of phenolic or aniline compounds, mediators and thus active intermediates such as free radicals and quinones are formed, which can further react with tetrameric compounds that laccase cannot directly oxidize [120]. Examples of efficient laccase mediators are ferulic acid, vanillin, and HBT [121]. Natural organic material, such as soybean meal, can be used as a mediator [29].

The combination of laccase and soybean meal as the mediator during 140 days in a soil slurry system resulted in a 40% degradation of PFOA and a 24% PFOA degradation in water after 36 days [29]. The used laccase was a purified laccase from *Pleurotus ostreatus* and a crude laccase solution concentrated from the fermentation broth of the fungus *Pycnoporus* sp. SYBC-L3. The authors pointed out that PFOA was effectively degraded by ECOHRs, with soybean meal as the mediator. Besides the soybean meal, the humate and mushroom compost was tested as natural mediators as well. The identified PFOA degradation products in the soil were partially fluorinated organic compounds containing perfluoroalkyl moieties. The proposed degradation mechanisms were free radical chain reaction processes that were started by direct free radical attacks on the C-C bonds in perfluoroalkyl acids and then fragmentation, rearrangement, and cross-coupling mechanisms [29]. Broman et al. [122] have used two-step treatment technology for the destruction of PFASs in water. The first step includes reverse osmosis and foam fractionation, and the second one includes laccase enzyme immobilized polyvinylidene fluoride membranes. The enzymes had a 35% efficiency rate for breaking down PFOA and PFOS. Qingguo [123] and Luo et al. [30] showed the degradation of PFOA via ECOHR. Qingguo [123] studied PFOA removal under different ECOHR conditions and achieved 30% PFOA degradation. Luo et al. [30] achieved 50% of PFOA degradation with laccase enzyme within 157 days. They detect partially fluorinated shorter-chain alcohols and aldehydes and no shorter carbon-chain PFCA as degradation products. Laccase-mediated ECOHR and granular activated carbon immobilized laccase accomplished over 25% PFOA reduction and 30% PFOA mineralization, confirming their potential for PFOA degradation [123]. 

The role and effectiveness of laccases in PFAS degradation have been reported by several studies [30,94,98] (described in more detail in the chapter Fungi in biodegradation/transformation of PFASs).

### 5.2. Peroxidases

Peroxidases can be found in plants, animals, microbes, bacteria, and fungi. Most peroxidases contain hem. To catalyze the oxidation of a wide range of inorganic and organic substrates, peroxidases use H_2_O_2_ or organic hydroperoxide as a cosubstrate. Peroxidases have high specificity, so they are used for efficient degradation of pollutants. An excessive amount of added H_2_O_2_ deactivates peroxidases. Peroxidase reactions are carried out when H_2_O_2_ reacts with an enzyme. The enzyme is oxidized, and a radical cation is formed—Compound I; and H_2_O_2_ is reduced to water. Then there is a reduction of Compound I, which oxidizes the organic substrate to Compound II and an organic radical. After that, Compound II oxidizes another organic molecule, whereby a new organic radical is formed, and the enzymes are reduced back to their resting form [124]. During the action of peroxidases, organic radicals are formed, which are responsible for the decomposition of pollutants [118]. The peroxidases CPO (chloroperoxidase), HRP, LiP, SBP (soybean peroxidase), and MnP are commonly used for the degradation of new pollutants and micropollutants [114]. Colosi et al. [93] achieved PFOA degradation with HRP, hydrogen peroxide, and a phenolic cosubstrate. Results indicated a 68% depletion of the parent compound in 6 h.

The degradation of PFASs by peroxidases was investigated in several studies [27,93,94], and the achieved results are described in the chapter Fungi in biodegradation/transformation of PFASs.

## 6. Conclusions

Per- and polyfluoroalkyl substances (PFASs), “forever chemicals”, are emerging contaminants classified as bioaccumulative in the environment due to high levels of molecular stability, recalcitrance, toxicity, and environmentally persistent. Recent studies in microbial degradation of PFASs have reported that an increasing number of PFAS species are biodegradable. Polyfluoroalkyl substances are more prone to microbial attack than perfluoroalkyl compounds. Bacteria, fungi, and enzymes show potential for degradation of PFAS species, but further investigation is still needed. The challenges in the PFAS degradation field are the optimization of the enzyme biosynthesis process; the enzyme activity, the scale-up from a lab- to full-scale application; the degradation mechanisms, the degradation genes, and gene modification. Furthermore, future investigations should include an investigation of the methods, technologies, processes, and parameters for PFAS detection/transformation/degradation. This review gives up-to-date information on recent findings in the field of PFAS biodegradation through bacterial and fungal degradation, as well as enzyme transformation/degradation.

## Data Availability

Not applicable.

## Abbreviations

6:2 FTS fluorotelomer sulfonate
6:2 FTSA 6:2 fluorotelomer sulfonic acid
AFFFs aqueous film-forming foams
BOD biological oxygen demand
CPO chloroperoxidase
ECOC enzyme-catalyzed oxidative coupling
ECOHRs enzyme catalysed oxidative humification reactions
ESI-HRMS Electrospray ionization-High-resolution mass spectrometry
EtFOSE N-ethyl perfluorooctane sulfonamidoethanol
FTOH fluorotelomer alcohol
GC-MS gas chromatography-mass spectrometry
HBT 1-hydroxybenzotriazole
HPLC high-performance liquid chromatograph
HR-MS high resolution mass spectrometry
HRP horseradish peroxidase
IC ion chromatography
LaC laccase
LC-MS/MS liquid chromatography-tandem mass spectrometry
LiP lignin peroxidase
MBRs membrane bioreactors
MnP manganese peroxidase
MS Mass Spectrometry
MS/MS Tandem Mass Spectrometry
OECD Organisation for Economic Co-operation and Development
PFAA perfluoroalkyl acids
PFASs perfluoroalkyl and polyfluoroalkyl substances
PFBS perfluorobutane sulfonate
PFCs perfluorocarbons
PFCA perfluoroalkyl carboxylic acid
PFHxA perfluorohexanoate
PFHxS perfluorohexane sulfonate
PFOA perfluorooctanoic acid
PFOS perfluorooctane sulfonic acid
PFPEs perfluoropolyethers
PFSA perfluoroalkyl sulfonic acid
PPOSA perfluoroalkyl sulfonamides
SBP soybean peroxidase
TCE trichloroethene
TFA trifluoroacetic acid
WWTP wastewater treatment plant
